# 6-Phenyl-6,7-dihydro­dibenzo[*c*,*f*][1,5]aza­bis­mocin-12(5*H*)-yl perchlorate

**DOI:** 10.1107/S1600536811021039

**Published:** 2011-06-11

**Authors:** Xiao-Wen Zhang, Ting Fan

**Affiliations:** a College of Environment Protection and Safety Engineering, University of South China, Hengyang 421001, People’s Republic of China; b Key Laboratory of Pollution Control and Resource Use of Hunan Province, University of South China, Hengyang 421001, People’s Republic of China

## Abstract

In the title compound, [Bi(C_20_H_17_N)(ClO_4_)] or C_20_H_17_BiClNO_4_, the Bi^III^ ion assumes a distorted ψ trigonal–bipyramidal geometry, with two C atoms and the electron lone pair of the Bi atom at the equatorial positions and an amine N atom and a perchlorate O atom at the apical positions. Weak inter­molecular C—H⋯O hydrogen bonding is present in the crystal structure.

## Related literature

For the synthesis of 12-chloro-6-phenyl-5,6,7,12-tetra­hydro­dibenzo[*c*,*f*][1,5]aza­bis­mocine, see: Zhang *et al.* (2009 ▶). For general background, see: Shimada *et al.* (2004 ▶); Yin *et al.* (2008 ▶); Zhang *et al.* (2010 ▶); Tan & Zhang (2011 ▶). For related structures, see: Ohkata *et al.* (1989 ▶); Minoura *et al.* (1999 ▶). 
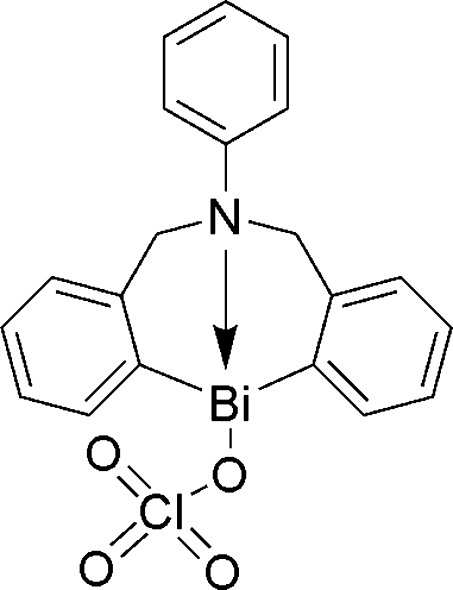


## Experimental

### 

#### Crystal data

[Bi(C_20_H_17_N)(ClO_4_)]
*M* *_r_* = 579.78
Monoclinic, 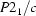

*a* = 12.0635 (10) Å
*b* = 14.0755 (12) Å
*c* = 11.5121 (10) Å
β = 107.590 (2)°
*V* = 1863.4 (3) Å^3^
*Z* = 4
Mo *K*α radiation
μ = 9.63 mm^−1^
*T* = 293 K
0.32 × 0.21 × 0.20 mm

#### Data collection

Bruker SMART 1000 CCD area-detector diffractometer
Absorption correction: multi-scan(*SADABS*; Bruker, 2001 ▶) *T*_min_ = 0.100, *T*_max_ = 0.145
9267 measured reflections
3279 independent reflections
2585 reflections with *I* > 2σ(*I*)
*R*_int_ = 0.166

#### Refinement

*R*[*F* ^2^ > 2σ(*F* ^2^)] = 0.065
*wR*(*F* ^2^) = 0.163
*S* = 1.02
3279 reflections
244 parameters
H-atom parameters constrained
Δρ_max_ = 4.89 e Å^−3^
Δρ_min_ = −4.14 e Å^−3^

### 

Data collection: *SMART* (Bruker, 2007 ▶); cell refinement: *SAINT* (Bruker, 2007 ▶); data reduction: *SAINT*; program(s) used to solve structure: *SHELXTL* (Sheldrick, 2008 ▶); program(s) used to refine structure: *SHELXTL*; molecular graphics: *SHELXTL*; software used to prepare material for publication: *SHELXTL*.

## Supplementary Material

Crystal structure: contains datablock(s) I, global. DOI: 10.1107/S1600536811021039/xu5208sup1.cif

Structure factors: contains datablock(s) I. DOI: 10.1107/S1600536811021039/xu5208Isup2.hkl

Supplementary material file. DOI: 10.1107/S1600536811021039/xu5208Isup3.cml

Additional supplementary materials:  crystallographic information; 3D view; checkCIF report

## Figures and Tables

**Table 1:** Selected bond lengths (Å)

Bi—N1	2.387 (10)
Bi—O1	2.546 (10)
Bi—C1	2.245 (13)
Bi—C8	2.204 (12)

**Table 2:** Hydrogen-bond geometry (Å, °)

*D*—H⋯*A*	*D*—H	H⋯*A*	*D*⋯*A*	*D*—H⋯*A*
C4—H4⋯O3^i^	0.93	2.46	3.137 (17)	130
C14—H14*B*⋯O2^ii^	0.97	2.55	3.398 (16)	146

Symmetry codes: (i) 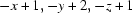; (ii) 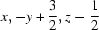.

**Fig. 1.**
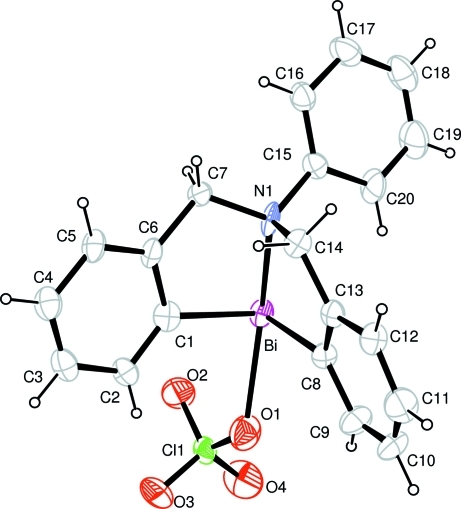
The molecular structure of the title compound with atomic numbering scheme. Displacement ellipsoids are drawn at the 30% probability level. All hydrogen atoms are omitted for clarity.

## supplementary crystallographic information

### Comment

Bismuth is a nontoxic and noncarcinogenic element and many of its compounds are low in toxicity and can be safely used in areas such as medicine, catalysis, and synthesis (Shimada *et al.*, 2004; Yin *et al.*, 2008; Zhang, Qiu, Tan *et al.*, 2010). The 5,6,7,12-tetrahydrodibenz[c,f][1,5]azabismocine framework is highly stable as a organobismuth Fragment because the weakly coordination exists between bismuth and nitrogen atoms on 1,5-azabismocine (Ohkata *et al.*,1989; Minoura *et al.*,1999), and therefore, is suitable for the study of organobismuth compounds bearing various groups on the bismuth and nitrogen atom for potential uses.

In the present paper, we report the crystal structure of the title compound (Fig. 1). The central bismuth–containing part of the complex exhibits a distorted pseudo trigonal–bipyramidal structure. The C (8), C (1) atoms and a lone electron pair of the Bi atom exist at the equatorial positions while the N (1) and O (1) atoms are located at the apical positions. The Bi–C (8) and Bi–C (1) distance is 2.250 (13) Å and 2.204 (12) Å, respectively. The C (8)–Bi–C (1) angle is 92.5 (5) ° while the N (1)–Bi–O (1) angle is 154.0 (3)°(rather than 180°). The Bi–N (1) distance (2.388 (10) Å) is shorter than 2.607 (5) Å of the precursor, C_6_H_5_N(CH_2_C_6_H_4_)_2_BiCl.The Bi–O (1) distance(2.546 (10) Å) is shorter than Bi–Cl(1) distance 2.597 (19) Å also(Zhang, Xia, Yan *et al.*, 2009).

### Experimental

The following procedures are recommended for synthesis of the title compound (I): 12-chloro-6-phenyl-5,6,7,12-tetrahydrodibenzo[c,f][1,5]azabismocine (0.516 g, 1.0 mmol) was dissolved in 15 ml THF, then a solution of AgClO_4 _(0.207 g, 1.0 mmol) in 15.0 ml THF was added. After the mixture was stirred in the dark at room temperature for 2 h, it was filtered. The filtrate mixed with 1.0 ml hexane was refrigerated for 24 h, giving colorless crystals (0.539 g, 93.0%).

### Refinement

All H atoms were positioned geometrically and refined using a riding model, with C-H = 0.93 Å for aryl, 0.98 Å methine and 0.97 Å for methylene H atoms, respectively. *U*_iso_(H)= 1.2*U*_eq_(C) for all H atoms.

### Crystal data

**Table:**

[Bi(C_20_H_17_N)(ClO_4_)]	*F*(000) = 1104
*M**_r_* = 579.78	*D*_x_ = 2.067 Mg m^−^^3^
Monoclinic, *P*2_1_/*c*	Mo *K*α radiation, λ = 0.71073 Å
Hall symbol: -P 2ybc	Cell parameters from 2936 reflections
*a* = 12.0635 (10) Å	θ = 2.7–24.2°
*b* = 14.0755 (12) Å	µ = 9.63 mm^−^^1^
*c* = 11.5121 (10) Å	*T* = 293 K
β = 107.590 (2)°	Prismatic, colorless
*V* = 1863.4 (3) Å^3^	0.32 × 0.21 × 0.20 mm
*Z* = 4	

### Data collection

**Table:**

Bruker SMART 1000 CCD area-detector diffractometer	3279 independent reflections
Radiation source: fine-focus sealed tube	2585 reflections with *I* > 2σ(*I*)
graphite	*R*_int_ = 0.166
φ and ω scans	θ_max_ = 25.0°, θ_min_ = 1.8°
Absorption correction: empirical (using intensity measurements) (*SADABS*; Bruker, 2001)	*h* = −10→14
*T*_min_ = 0.100, *T*_max_ = 0.145	*k* = −13→16
9267 measured reflections	*l* = −13→13

### Refinement

**Table:**

Refinement on *F*^2^	Primary atom site location: structure-invariant direct methods
Least-squares matrix: full	Secondary atom site location: difference Fourier map
*R*[*F*^2^ > 2σ(*F*^2^)] = 0.065	Hydrogen site location: inferred from neighbouring sites
*wR*(*F*^2^) = 0.163	H-atom parameters constrained
*S* = 1.02	*w* = 1/[σ^2^(*F*_o_^2^) + (0.0816*P*)^2^] where *P* = (*F*_o_^2^ + 2*F*_c_^2^)/3
3279 reflections	(Δ/σ)_max_ = 0.001
244 parameters	Δρ_max_ = 4.89 e Å^−^^3^
0 restraints	Δρ_min_ = −4.14 e Å^−^^3^

### Special details

**Table:**

Geometry. All esds (except the esd in the dihedral angle between two l.s. planes) are estimated using the full covariance matrix. The cell esds are taken into account individually in the estimation of esds in distances, angles and torsion angles; correlations between esds in cell parameters are only used when they are defined by crystal symmetry. An approximate (isotropic) treatment of cell esds is used for estimating esds involving l.s. planes.
Refinement. Refinement of F^2^ against ALL reflections. The weighted R-factor wR and goodness of fit S are based on F^2^, conventional R-factors R are based on F, with F set to zero for negative F^2^. The threshold expression of F^2^ > 2sigma(F^2^) is used only for calculating R-factors(gt) etc. and is not relevant to the choice of reflections for refinement. R-factors based on F^2^ are statistically about twice as large as those based on F, and R- factors based on ALL data will be even larger.

### Fractional atomic coordinates and isotropic or equivalent isotropic displacement parameters (Å^2^)

**Table:**

	*x*	*y*	*z*	*U*_iso_*/*U*_eq_	
Bi	0.74646 (4)	0.77309 (3)	0.84604 (4)	0.0321 (2)	
Cl1	0.7240 (3)	1.0213 (2)	0.8825 (3)	0.0378 (7)	
N1	0.7225 (7)	0.6171 (8)	0.7606 (7)	0.033 (2)	
O1	0.7940 (9)	0.9494 (7)	0.8480 (10)	0.063 (3)	
O2	0.6291 (9)	0.9730 (7)	0.9075 (10)	0.069 (3)	
O3	0.6836 (10)	1.0854 (8)	0.7850 (10)	0.071 (3)	
O4	0.7937 (12)	1.0687 (8)	0.9874 (10)	0.086 (4)	
C1	0.6226 (11)	0.7895 (8)	0.6574 (11)	0.037 (3)	
C2	0.6002 (10)	0.8714 (8)	0.5878 (10)	0.034 (3)	
H2	0.6373	0.9281	0.6181	0.041*	
C3	0.5204 (11)	0.8671 (9)	0.4706 (11)	0.041 (3)	
H3	0.5026	0.9222	0.4241	0.049*	
C4	0.4688 (12)	0.7846 (8)	0.4240 (12)	0.042 (3)	
H4	0.4163	0.7830	0.3459	0.051*	
C5	0.4949 (11)	0.7005 (9)	0.4943 (11)	0.037 (3)	
H5	0.4603	0.6435	0.4617	0.045*	
C6	0.5695 (10)	0.7026 (9)	0.6080 (10)	0.033 (3)	
C7	0.5996 (10)	0.6133 (8)	0.6827 (9)	0.031 (3)	
H7A	0.5489	0.6068	0.7335	0.037*	
H7B	0.5881	0.5586	0.6292	0.037*	
C8	0.8978 (11)	0.7594 (8)	0.7776 (11)	0.030 (3)	
C9	0.9896 (11)	0.8252 (10)	0.7942 (12)	0.047 (3)	
H9	0.9914	0.8787	0.8421	0.056*	
C10	1.0741 (11)	0.8122 (10)	0.7424 (14)	0.051 (4)	
H10	1.1337	0.8565	0.7542	0.062*	
C11	1.0723 (14)	0.7321 (10)	0.6709 (16)	0.055 (4)	
H11	1.1291	0.7246	0.6321	0.066*	
C12	0.9893 (10)	0.6651 (9)	0.6570 (11)	0.038 (3)	
H12	0.9919	0.6101	0.6132	0.045*	
C13	0.8986 (9)	0.6785 (8)	0.7091 (9)	0.030 (2)	
C14	0.8015 (10)	0.6065 (8)	0.6812 (10)	0.033 (3)	
H14A	0.8350	0.5433	0.6923	0.040*	
H14B	0.7555	0.6128	0.5965	0.040*	
C15	0.7472 (9)	0.5399 (9)	0.8534 (10)	0.033 (3)	
C16	0.6852 (11)	0.4552 (9)	0.8367 (12)	0.041 (3)	
H16	0.6240	0.4455	0.7659	0.049*	
C17	0.7139 (13)	0.3865 (10)	0.9238 (14)	0.053 (4)	
H17	0.6723	0.3298	0.9117	0.064*	
C18	0.8062 (14)	0.4002 (11)	1.0325 (14)	0.058 (4)	
H18	0.8251	0.3532	1.0919	0.069*	
C19	0.8671 (14)	0.4831 (12)	1.0489 (13)	0.064 (4)	
H19	0.9281	0.4920	1.1202	0.077*	
C20	0.8408 (11)	0.5534 (11)	0.9634 (11)	0.050 (3)	
H20	0.8833	0.6097	0.9763	0.060*	

### Atomic displacement parameters (Å^2^)

**Table:**

	*U*^11^	*U*^22^	*U*^33^	*U*^12^	*U*^13^	*U*^23^
Bi	0.0313 (3)	0.0438 (3)	0.0201 (3)	0.00040 (18)	0.0062 (2)	−0.00470 (18)
Cl1	0.0407 (17)	0.0414 (16)	0.0248 (14)	0.0024 (13)	0.0001 (12)	0.0037 (13)
N1	0.018 (5)	0.070 (7)	0.007 (4)	0.002 (4)	−0.001 (4)	0.001 (4)
O1	0.047 (6)	0.064 (7)	0.079 (8)	0.005 (5)	0.020 (5)	−0.009 (6)
O2	0.054 (6)	0.076 (7)	0.086 (8)	0.001 (5)	0.036 (6)	0.026 (6)
O3	0.082 (8)	0.065 (7)	0.059 (7)	0.000 (6)	0.008 (6)	0.034 (6)
O4	0.122 (11)	0.076 (8)	0.035 (6)	−0.017 (7)	−0.015 (6)	−0.011 (6)
C1	0.038 (7)	0.045 (7)	0.033 (7)	0.005 (5)	0.016 (6)	−0.005 (6)
C2	0.041 (7)	0.038 (6)	0.025 (6)	0.005 (5)	0.012 (5)	0.003 (5)
C3	0.043 (7)	0.053 (8)	0.032 (7)	0.013 (6)	0.019 (6)	0.013 (6)
C4	0.044 (8)	0.049 (8)	0.030 (7)	0.006 (6)	0.006 (6)	−0.004 (6)
C5	0.034 (7)	0.050 (8)	0.022 (6)	0.002 (5)	−0.002 (5)	−0.008 (5)
C6	0.021 (6)	0.054 (7)	0.025 (6)	0.000 (5)	0.006 (5)	0.007 (5)
C7	0.033 (6)	0.036 (6)	0.020 (5)	−0.006 (5)	0.003 (5)	0.004 (5)
C8	0.030 (7)	0.038 (6)	0.027 (6)	0.001 (5)	0.015 (5)	0.003 (5)
C9	0.045 (8)	0.045 (8)	0.048 (8)	−0.013 (6)	0.010 (6)	−0.015 (7)
C10	0.031 (7)	0.058 (8)	0.066 (9)	−0.013 (6)	0.015 (7)	0.005 (8)
C11	0.051 (10)	0.066 (10)	0.058 (10)	0.007 (7)	0.030 (8)	0.012 (8)
C12	0.035 (7)	0.046 (7)	0.034 (6)	0.004 (5)	0.012 (5)	−0.003 (6)
C13	0.031 (6)	0.039 (6)	0.020 (5)	0.002 (5)	0.006 (5)	0.003 (5)
C14	0.034 (6)	0.042 (7)	0.026 (6)	−0.001 (5)	0.014 (5)	−0.003 (5)
C15	0.036 (7)	0.036 (7)	0.030 (6)	0.004 (5)	0.015 (5)	0.006 (5)
C16	0.038 (7)	0.045 (8)	0.039 (7)	0.005 (6)	0.010 (6)	0.010 (6)
C17	0.059 (9)	0.045 (8)	0.063 (9)	0.005 (7)	0.029 (8)	0.020 (7)
C18	0.066 (10)	0.063 (10)	0.050 (9)	0.020 (8)	0.026 (8)	0.024 (8)
C19	0.061 (10)	0.083 (12)	0.041 (8)	0.011 (9)	0.005 (7)	0.018 (8)
C20	0.042 (8)	0.069 (10)	0.027 (7)	0.000 (7)	−0.007 (6)	0.004 (7)

### Geometric parameters (Å, °)

**Table:**

Bi—N1	2.387 (10)	C8—C13	1.387 (17)
Bi—O1	2.546 (10)	C8—C9	1.411 (17)
Bi—C1	2.245 (13)	C9—C10	1.340 (18)
Bi—C8	2.204 (12)	C9—H9	0.9300
Cl1—O3	1.407 (10)	C10—C11	1.39 (2)
Cl1—O4	1.413 (10)	C10—H10	0.9300
Cl1—O2	1.434 (10)	C11—C12	1.349 (19)
Cl1—O1	1.448 (10)	C11—H11	0.9300
N1—C7	1.484 (13)	C12—C13	1.410 (16)
N1—C15	1.490 (15)	C12—H12	0.9300
N1—C14	1.514 (12)	C13—C14	1.508 (15)
C1—C2	1.383 (16)	C14—H14A	0.9700
C1—C6	1.418 (17)	C14—H14B	0.9700
C2—C3	1.402 (17)	C15—C16	1.389 (18)
C2—H2	0.9300	C15—C20	1.432 (16)
C3—C4	1.351 (18)	C16—C17	1.360 (18)
C3—H3	0.9300	C16—H16	0.9300
C4—C5	1.414 (18)	C17—C18	1.41 (2)
C4—H4	0.9300	C17—H17	0.9300
C5—C6	1.347 (16)	C18—C19	1.36 (2)
C5—H5	0.9300	C18—H18	0.9300
C6—C7	1.504 (16)	C19—C20	1.365 (19)
C7—H7A	0.9700	C19—H19	0.9300
C7—H7B	0.9700	C20—H20	0.9300
			
C8—Bi—C1	92.5 (5)	C13—C8—C9	118.4 (11)
C8—Bi—N1	77.4 (4)	C13—C8—Bi	115.1 (8)
C1—Bi—N1	74.6 (4)	C9—C8—Bi	126.5 (9)
C8—Bi—O1	83.1 (4)	C10—C9—C8	121.5 (13)
C1—Bi—O1	89.4 (4)	C10—C9—H9	119.3
N1—Bi—O1	154.0 (3)	C8—C9—H9	119.3
O3—Cl1—O4	110.7 (7)	C9—C10—C11	119.7 (13)
O3—Cl1—O2	111.0 (7)	C9—C10—H10	120.2
O4—Cl1—O2	110.9 (7)	C11—C10—H10	120.2
O3—Cl1—O1	108.6 (7)	C12—C11—C10	120.9 (13)
O4—Cl1—O1	108.6 (8)	C12—C11—H11	119.5
O2—Cl1—O1	106.9 (6)	C10—C11—H11	119.5
C7—N1—C15	110.7 (9)	C11—C12—C13	120.0 (12)
C7—N1—C14	109.2 (8)	C11—C12—H12	120.0
C15—N1—C14	109.6 (8)	C13—C12—H12	120.0
C7—N1—Bi	104.8 (7)	C8—C13—C12	119.4 (11)
C15—N1—Bi	113.7 (6)	C8—C13—C14	122.3 (10)
C14—N1—Bi	108.6 (7)	C12—C13—C14	118.2 (10)
Cl1—O1—Bi	122.3 (6)	C13—C14—N1	113.3 (9)
C2—C1—C6	120.1 (12)	C13—C14—H14A	108.9
C2—C1—Bi	127.1 (9)	N1—C14—H14A	108.9
C6—C1—Bi	112.7 (8)	C13—C14—H14B	108.9
C1—C2—C3	118.6 (12)	N1—C14—H14B	108.9
C1—C2—H2	120.7	H14A—C14—H14B	107.7
C3—C2—H2	120.7	C16—C15—C20	119.0 (11)
C4—C3—C2	121.3 (12)	C16—C15—N1	123.0 (10)
C4—C3—H3	119.4	C20—C15—N1	118.0 (11)
C2—C3—H3	119.4	C17—C16—C15	120.1 (13)
C3—C4—C5	119.8 (13)	C17—C16—H16	119.9
C3—C4—H4	120.1	C15—C16—H16	119.9
C5—C4—H4	120.1	C16—C17—C18	120.8 (14)
C6—C5—C4	120.4 (12)	C16—C17—H17	119.6
C6—C5—H5	119.8	C18—C17—H17	119.6
C4—C5—H5	119.8	C19—C18—C17	119.2 (13)
C5—C6—C1	119.8 (12)	C19—C18—H18	120.4
C5—C6—C7	120.9 (11)	C17—C18—H18	120.4
C1—C6—C7	119.3 (11)	C18—C19—C20	121.6 (15)
N1—C7—C6	109.9 (9)	C18—C19—H19	119.2
N1—C7—H7A	109.7	C20—C19—H19	119.2
C6—C7—H7A	109.7	C19—C20—C15	119.4 (14)
N1—C7—H7B	109.7	C19—C20—H20	120.3
C6—C7—H7B	109.7	C15—C20—H20	120.3
H7A—C7—H7B	108.2		

### Hydrogen-bond geometry (Å, °)

**Table:**

*D*—H···*A*	*D*—H	H···*A*	*D*···*A*	*D*—H···*A*
C4—H4···O3^i^	0.93	2.46	3.137 (17)	130
C14—H14B···O2^ii^	0.97	2.55	3.398 (16)	146

Symmetry codes: (i) −*x*+1, −*y*+2, −*z*+1; (ii) *x*, −*y*+3/2, *z*−1/2.
